# Comparative Evaluation of Salivary Chemerin and Malondialdehyde Among Patients With Periodontitis and Oral Squamous Cell Carcinoma: A Cross-Sectional Study

**DOI:** 10.7759/cureus.111974

**Published:** 2026-07-03

**Authors:** Bhavana J Jadhav, Siddhartha Varma, Girish Suragimath, Sameer A Zope, Vaishali N Mashalkar, Apurva V Kale

**Affiliations:** 1 Department of Periodontology, School of Dental Sciences, Krishna Vishwa Vidyapeeth (Deemed to be University), Karad, IND

**Keywords:** chemerin, elisa, malondialdehyde, oral squamous cell carcinoma, periodontitis

## Abstract

Introduction

Periodontitis and oral squamous cell carcinoma (OSCC) are long-lasting inflammatory conditions that lead to tissue damage, oxidative stress, and changes in immune responses. Recent studies indicate a potential biological link between periodontal inflammation and the development of oral cancer. Salivary biomarkers such as chemerin and malondialdehyde (MDA) may serve as non-invasive tools for early diagnosis and disease monitoring. This study was designed to assess and compare salivary chemerin and MDA levels in three groups: healthy individuals, patients with periodontitis, and those with OSCC.

Methods

This study was conducted on 75 participants distributed evenly among the three groups: healthy individuals (Group A), periodontitis patients (Group B), and OSCC patients (Group C). Clinical periodontal parameters, including Oral Hygiene Index-Simplified (OHI-S), Russell's periodontal index, probing pocket depth (PPD), and clinical attachment level (CAL), were recorded. Unstimulated saliva samples were collected and analyzed for chemerin and MDA levels using enzyme-linked immunosorbent assay (ELISA) kits. Statistical analysis was done using analysis of variance (ANOVA), Tukey's post hoc test, and Pearson's correlation coefficient, with p<0.05 considered statistically significant.

Results

Salivary chemerin and MDA levels showed a progressive increase from healthy controls to periodontitis patients and OSCC patients. One-way ANOVA revealed statistically significant differences among the three groups for both chemerin (F(2,72)=4.28; p=0.017) and MDA (F(2,72)=3.45; p=0.037). Tukey's post hoc analysis demonstrated significant pairwise differences between all groups for both biomarkers (p<0.001), indicating increasing levels with disease severity. Pearson's correlation analysis showed a statistically significant positive correlation between chemerin and MDA, suggesting an association between inflammatory and oxidative stress pathways in periodontal disease.

Conclusion

Salivary chemerin and MDA levels were significantly increased in periodontitis and OSCC patients, with the highest levels observed in OSCC. These findings suggest that chemerin and MDA may serve as promising non-invasive salivary biomarkers for the diagnosis, risk assessment, and monitoring of periodontal disease and OSCC. Further large-scale longitudinal studies are recommended to validate their clinical applicability.

## Introduction

Oral health is closely linked to overall systemic health, with conditions like periodontitis and oral squamous cell carcinoma (OSCC) reflecting or influencing broader disease processes [1]. Both conditions are characterized by chronic inflammation, tissue destruction, and biochemical alterations in the oral environment [2]. Emerging studies suggest a biological link between periodontal inflammation and oral carcinogenesis, highlighting the potential of salivary biomarkers as non-invasive tools for early diagnosis and monitoring [3].

Periodontal disease is a long-term inflammatory disease that affects the tooth-supporting structures, driven by microbial biofilm and the host immune response [4]. In addition to causing localized harm, it plays a role in systemic inflammation and is linked to various diseases, including diabetes, cardiovascular issues, and cancer [5]. OSCC, the most commonly occurring oral cancer, is especially common in developing nations, largely due to factors like tobacco use and inadequate oral hygiene. Unfortunately, it is often detected at advanced stages, with chronic inflammation being a well-known factor in its development [6].

Persistent inflammation in periodontitis may encourage cancer development through oxidative stress, cytokine release, DNA damage, and the activation of oncogenic pathways like NF-κB [7]. Exploring shared biomarkers could improve understanding and aid in early detection. Saliva, being non-invasive and reflective of both local and systemic health, is ideal for biomarker analysis [8]. Chemerin, an inflammation-related protein, and malondialdehyde (MDA), a marker of oxidative stress, have shown potential in this regard [9,10].

The goal of this study was to assess and compare salivary levels of chemerin and MDA among healthy individuals, periodontitis patients, and those with OSCC, to assess their utility as diagnostic biomarkers.

## Materials and methods

Study design and ethical approval

A cross-sectional study was conducted in the Department of Periodontology, School of Dental Sciences, Krishna Vishwa Vidyapeeth (Deemed to be University), Karad, India. Ethical approval for the study was obtained from the Institutional Ethics Committee of Krishna Institute of Medical Sciences (Deemed to be University) (approval number: KIMSDU/IEC/06/2023). The study was conducted in accordance with the ethical principles of the Declaration of Helsinki.

Sample size and sampling technique

A total of 75 participants were included in the study using stratified random sampling. All individuals attending the outpatient department were screened for eligibility and underwent a comprehensive periodontal examination. The sample size was determined using power analysis, which indicated that 75 participants (25 in each group) would provide 95% power to detect significant differences among the groups at the 5% significance level (p<0.05).

The sample size is calculated using the following formula: $n=\frac{(Z_{\alpha}+Z_{\beta})^{2}\times\left(S_{1}^{2}+S_{2}^{2}\right)}{(m_{2}-m_{1})^{2}}$. Here, n is the sample size per group, $Z_{\alpha}$​ is the standard normal deviate for the significance level, $Z_{\beta}$ is the standard normal deviate for the desired power, $S_{1}^{2}$​ and $S_{2}^{2}$ are the variances of Groups 1 and 2, and $m_{1}$ and $m_{2}$ are the means of Groups 1 and 2.

Inclusion criteria

Individuals who provided written informed consent and were clinically diagnosed with periodontal disease according to the 2017 World Workshop Classification of Periodontal and Peri-implant Diseases and Conditions [11] were included in the study. Based on clinical findings, participants were categorized into three groups: Group A, periodontally healthy individuals; Group B, patients with periodontitis (stages II, III, and IV); and Group C, patients diagnosed with OSCC with or without periodontitis. 

Exclusion criteria

Participants were excluded if they had any history of systemic diseases, were pregnant or lactating, had undergone periodontal therapy within the past three months, were on antibiotics or anti-inflammatory or antiplatelet medications, or had a history of smoking or tobacco use.

Periodontal clinical examination

Clinical evaluations included the Simplified Oral Hygiene Index (OHI-S) developed by Greene and Vermillion [12] and Russell's periodontal index developed by Russell [13]. Both indices are standardized clinical assessment tools that are available in the public domain and do not require licensing or permission for research use. Probing pocket depth (PPD) and clinical attachment level (CAL) were recorded using standard periodontal examination procedures.

Saliva sample collection

Unstimulated whole saliva samples (2 mL) were collected from each participant between 10:00 AM and 12:00 PM using a modified method described by Navazesh [14]. Participants were instructed to refrain from eating, drinking, smoking, or performing oral hygiene procedures for at least one hour prior to sample collection. Clinical examination was completed at least 60 minutes before saliva collection to avoid contamination. Participants were asked to swallow initially and then allow saliva to accumulate passively in the floor of the mouth and expectorate into sterile tubes over a five-minute period.

Saliva processing and storage

Collected saliva samples were centrifuged at 1000 rpm for 15 minutes at 2-8°C to remove debris and cellular components. The supernatant was aliquoted and stored at -80°C until biochemical analysis. Samples were analyzed within two months of collection to ensure the stability of biomarkers.

Biochemical analysis

Salivary levels of chemerin and MDA were quantified using commercially available enzyme-linked immunosorbent assay (ELISA) kits (ELK Biotechnology Co. Ltd., Wuhan, China), following the manufacturer's instructions. All analyses were performed in the Microbiology Laboratory of Krishna Vishwa Vidyapeeth (Deemed to be University), Karad, India.

Detection of human chemerin levels in saliva

Human CHEM (chemerin) ELISA kit (ELK Biotechnology Co. Ltd.; Catalog Number ELK1953 96) was used in the current study. The kit has a sensitivity of 0.069 ng/mL and a detection range of 0.16-10 ng/mL. The test principle applied in this kit is a sandwich enzyme immunoassay. The microtiter plate provided in this kit has been pre-coated with an antibody specific to CHEM. Standards or samples are added to the appropriate microtiter plate wells and then with a biotin-conjugated antibody specific to CHEM. Next, avidin conjugated to horseradish peroxidase (HRP) is added to each microplate well and incubated. After the TMB substrate solution is added, only those wells that contain CHEM, biotin-conjugated antibody, and enzyme-conjugated avidin will exhibit a change in color. The enzyme-substrate reaction is terminated by the addition of sulfuric acid solution, and the color change is measured spectrophotometrically at a wavelength of 450 nm±10 nm. The concentration of CHEM in the samples is then determined by comparing the optical density of the samples to the standard curve.

Detection of human MDA levels in saliva

Salivary MDA levels were quantified using a commercially available ELISA kit (ELK Biotechnology Co. Ltd.), following the manufacturer's instructions. The assay employs a competitive inhibition enzyme immunoassay technique. The microtiter plate provided in this kit has been pre-coated with MDA protein. Standards or samples are added to the appropriate microtiter plate wells along with a biotin-conjugated antibody specific to MDA. Next, avidin conjugated to HRP is added to each microplate well and incubated. After incubation, the TMB substrate solution is added, resulting in a color change. The enzyme-substrate reaction is terminated by the addition of sulfuric acid solution, and the color intensity is measured spectrophotometrically at a wavelength of 450 nm±10 nm. The concentration of MDA in the samples is then determined by comparing the optical density of the samples to the standard curve.

Statistical analysis

Data were analyzed using IBM SPSS Statistics for Windows, Version 21.0 (IBM Corp., Armonk, New York, United States). Descriptive statistics were expressed as mean±standard deviation. Intergroup comparisons were performed using one-way analysis of variance (ANOVA). Post hoc multiple comparisons were carried out using Tukey's test to identify pairwise differences among the groups. Pearson's correlation coefficient was used to assess relationships between biochemical and clinical parameters. A p-value of <0.05 was considered statistically significant.

## Results

Seventy-five participants were divided into three separate categories: Group A included 25 healthy individuals, Group B comprised 25 patients suffering from periodontitis, and Group C contained 25 individuals diagnosed with OSCC.

The analysis revealed a significant difference in the mean age across the three groups (F=3.19; p=0.047). Specifically, Group A had a mean age of 40.68±12.0 years (SE=2.4; range: 28.68-52.68), while Group B, which included periodontitis patients, had an older mean age of 48.4±8.77 years (SE=1.75; range: 39.63-57.17). Group C, containing patients with OSCC, displayed a mean age of 44.84±11.4 years (SE=2.28; range: 33.44-56.24) (Table 1).

**Table 1:** Age distribution of the participants *: statistically significant

Groups	Mean	SD	SE	Minimum	Maximum	F-value	P-value
Group A	40.68	12.0	2.4	28.68	52.68	3.19	0.047*
Group B	48.4	8.77	1.75	39.63	57.17		
Group C	44.84	11.4	2.28	33.44	56.24		

The groups exhibited a comparable gender distribution: Group A included 60% males and 40% females, while Groups B and C each consisted of 56% males and 44% females. The variation in gender distribution was not statistically significant (p=0.94) (Table 2).

**Table 2:** Gender distribution of the participants

Groups	Male	Female	Total	P-value
Group A	15 (60)	10 (40)	25 (100)	0.94
Group B	14 (56)	11 (44)	25 (100)	
Group C	14 (56)	11 (44)	25 (100)	

A clinical examination was conducted to compare periodontal parameters, including the OHI-S, Russell's periodontal index, PPD, and CAL, among the three groups. The analysis was performed using the ANOVA test, which indicated significant differences among the groups (p=0.000). Group A exhibited the best periodontal health, evidenced by significantly lower mean values in all evaluated indices: OHI-S=0.526, Russell's periodontal index=0.139, PPD=0, and CAL=0. In contrast, Group B demonstrated the most severe periodontal conditions, with higher scores, namely, OHI-S=3.613, Russell's periodontal index=3.51, PPD=7.42, and CAL=3.97, while Group C showed intermediate values, that is, OHI-S=2.397, Russell's periodontal index=2.852, PPD=6.45, and CAL=3.81. Notably, Group B consistently recorded the poorest outcomes, reflecting more advanced periodontal issues (Table 3).

**Table 3:** Analysis of variance test for periodontal clinical parameters *: statistically significant

Parameters	Groups	Mean	Std. deviation	Std. error	95% CI lower bound	95% CI upper bound	Minimum	Maximum	F-value	df	P-value
Oral Hygiene Index-Simplified	Group A	0.526	0.2190	0.0393	0.445	0.606	0.1	0.8	402.91	2,72	<0.001*
	Group B	3.613	0.4731	0.0850	3.439	3.786	2.9	5.2			
	Group C	2.397	0.4223	0.0758	2.242	2.552	1.5	3.2			
Russell's periodontal index	Group A	0.139	0.0667	0.0120	0.114	0.163	0.0	0.2	457.58	2,72	<0.001*
	Group B	3.510	0.3487	0.0626	3.382	3.638	2.7	4.2			
	Group C	2.852	0.6303	0.1132	2.620	3.083	1.8	4.0			
Probing pocket depth	Group A	0.00	0.000	0.000	0.00	0.00	0	0	809.62	2,72	<0.001*
	Group B	7.42	0.807	0.145	7.12	7.72	6	9			
	Group C	6.45	0.925	0.166	6.11	6.79	5	8			
Clinical attachment level	Group A	0.00	0.000	0.000	0.00	0.00	0	0	172.67	2,72	<0.001*
	Group B	3.97	0.875	0.157	3.65	4.29	3	6			
	Group C	3.81	1.195	0.215	3.37	4.24	0	6			

Following a significant ANOVA result, Tukey's post hoc test revealed statistically significant pairwise differences across all variables: OHI-S, Russell's periodontal index, PPD, and CAL. Group A consistently showed better periodontal health compared to Groups B and C, with significantly lower scores across all parameters (p<0.001). Group B exhibited the poorest clinical outcomes, followed by Group C. Specifically, OHI-S and Russell's periodontal index were lowest in Group A and highest in Group B, indicating poorer oral hygiene and greater periodontal destruction. PPD was shallowest in Group A and deepest in Group B, while CAL was significantly higher in Groups B and C compared to Group A, with no significant difference between Groups B and C. These findings demonstrate a progressive decline in periodontal status from healthy individuals to those with periodontitis and OSCC, highlighting the link between chronic inflammation and tissue breakdown (Table 4).

**Table 4:** Tukey's post hoc test for periodontal clinical parameters *: statistically significant Overall group comparisons were performed using one-way analysis of variance. OHI-S: F(2,72)=402.91; p<0.001. Russell's periodontal index: F(2,72)=457.58; p<0.001. Probing pocket depth: F(2,72)=809.62; p<0.001. Clinical attachment level: F(2,72)=172.67; p<0.001. Pairwise comparisons were performed using Tukey's post hoc test.

Dependent variable	Group	Group	Mean difference	Std. error	P-value	95% CI lower bound	95% CI upper bound
Oral Hygiene Index-Simplified	Group A	Group B	-3.087	0.095	<0.0001*	-3.38	-2.79
	Group A	Group C	-1.871	0.095	<0.0001*	-2.17	-1.57
	Group B	Group C	1.216	0.095	<0.0001*	0.92	1.51
Russell's periodontal index	Group A	Group B	-3.371	0.078	<0.0001*	-3.63	-3.11
	Group A	Group C	-2.713	0.078	<0.0001*	-2.97	-2.46
	Group B	Group C	0.658	0.078	0.002*	0.39	0.92
Probing pocket depth	Group A	Group B	-7.42	0.14	<0.0001*	-7.80	-7.04
	Group A	Group C	-6.45	0.14	<0.0001*	-6.83	-6.07
	Group B	Group C	0.97	0.14	0.025*	0.59	1.35
Clinical attachment level	Group A	Group B	-3.97	0.16	<0.0001*	-4.37	-3.57
	Group A	Group C	-3.81	0.16	<0.0001*	-4.21	-3.41
	Group B	Group C	0.16	0.16	0.965	-0.24	0.56

Pearson's correlation analysis was performed to evaluate the relationship between periodontal clinical parameters and salivary biomarkers (chemerin and MDA). Chemerin showed weak to moderate, non-significant positive correlations with OHI-S, Russell's periodontal index, PPD, and CAL (p>0.05).

A statistically significant positive correlation was observed between chemerin and MDA (r=0.996; p=0.036), suggesting a strong association between inflammatory activity and oxidative stress. MDA also showed moderate, non-significant positive correlations with periodontal clinical parameters (p>0.05) (Table 5).

**Table 5:** Pearson's correlation coefficient (r) was used to assess associations between variables *: statistically significant Only chemerin vs. malondialdehyde showed statistical significance (p=0.036).

Variables	Oral Hygiene Index-Simplified	Russell's periodontal Index	Probing pocket depth	Clinical attachment level	Chemerin	Malondialdehyde
Oral Hygiene Index-Simplified	1	-	-	-	0.261	0.351
Russell's periodontal index	-	1	-	-	0.459	0.538
Probing pocket depth	-	-	1	-	0.520	0.594
Clinical attachment level	-	-	-	1	0.592	0.658
Chemerin	0.261	0.459	0.520	0.592	1	0.996*
Malondialdehyde	0.351	0.538	0.594	0.658	0.996*	1

**Figure 1:**
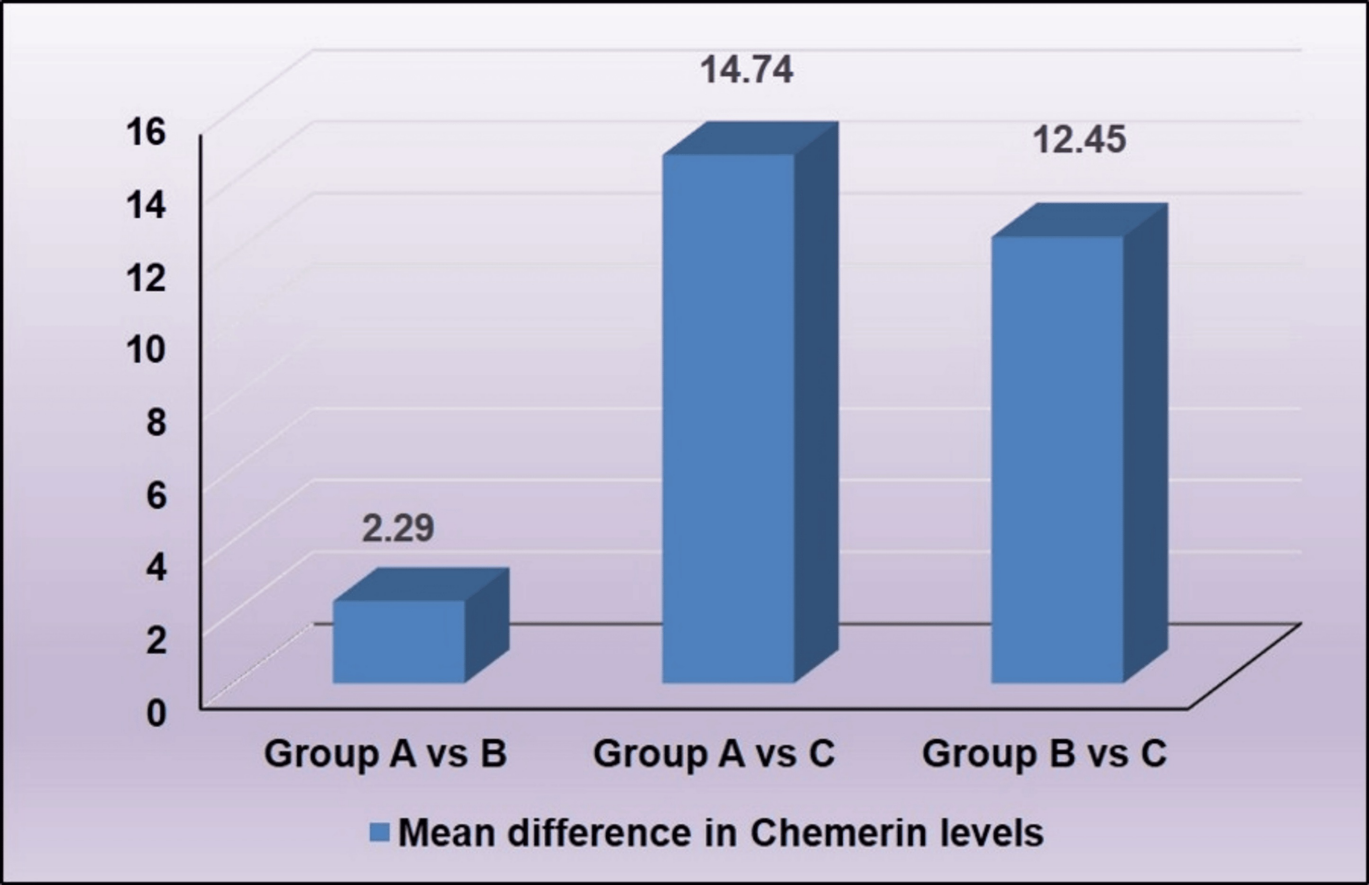
Comparison of mean salivary levels of chemerin between the three groups by Tukey's post hoc test

One-way ANOVA demonstrated a statistically significant difference in mean salivary chemerin levels among the three study groups (F(2,72)=4.28; p=0.017). Tukey's post hoc test revealed significant differences between all groups. Group A showed significantly lower chemerin levels compared to Groups B and C, while Group B also had significantly lower levels than Group C (p<0.0001 for all comparisons), indicating a progressive increase in salivary chemerin levels from Group A to Group C (Table 6 and Figure 1). 

**Table 6:** Tukey's post hoc test for pairwise comparison for chemerin *: statistically significant Overall comparison was performed using one-way analysis of variance: F(2,72)=4.28; p=0.017. Pairwise comparisons were performed using Tukey's post hoc test.

Comparison	Mean difference	P-value
Group A vs. Group B	-2.29	<0.0001*
Group A vs. Group C	-14.74	<0.0001*
Group B vs. Group C	-12.45	<0.0001*

**Figure 2:**
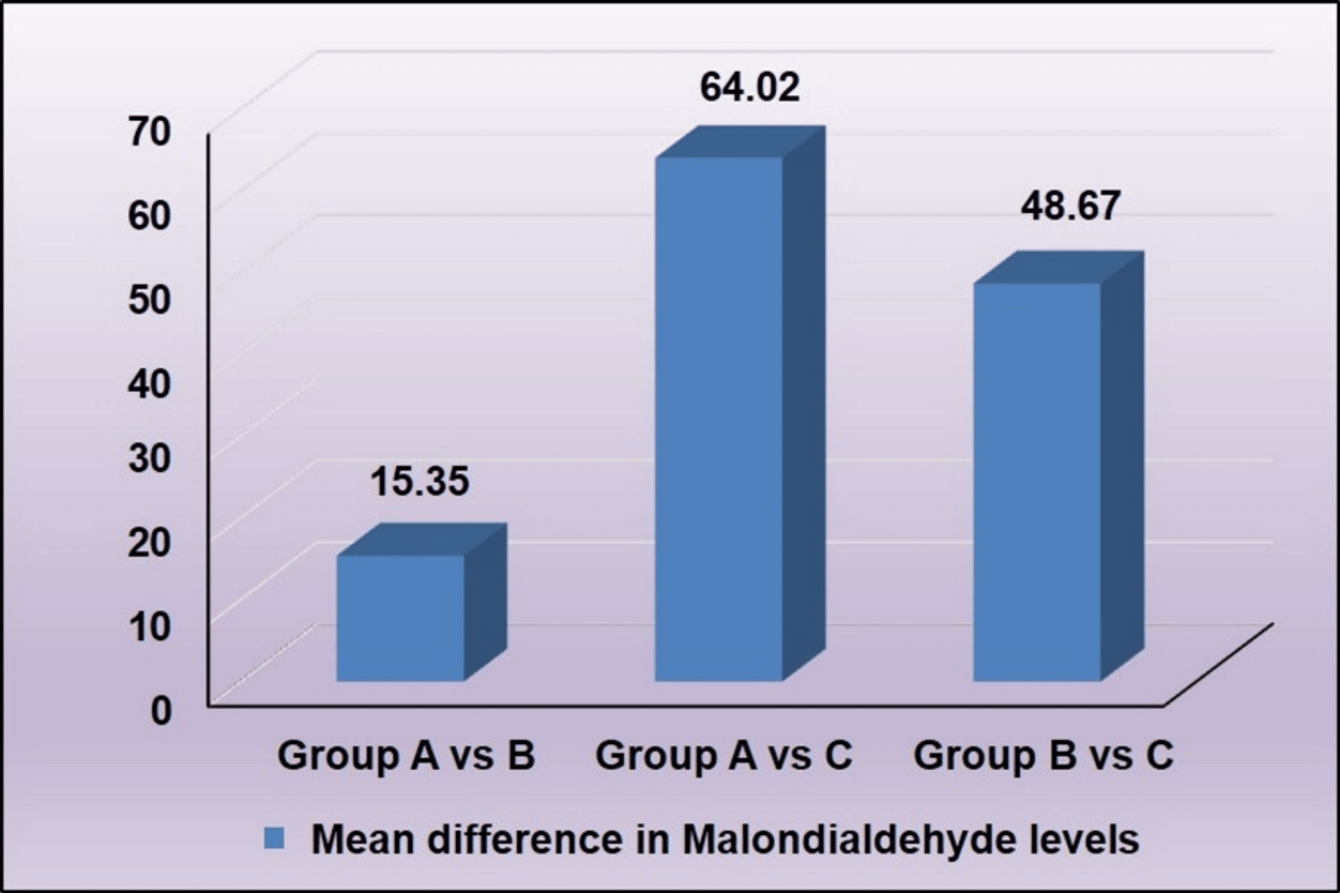
Comparison of mean salivary levels of malondialdehyde between the three groups by Tukey's post hoc test

One-way ANOVA revealed a statistically significant difference in mean salivary MDA levels among the three study groups (F(2,72)=3.45; p=0.037). Tukey's post hoc analysis demonstrated significantly higher MDA levels in Group C compared with Groups A and B, while Group B also exhibited significantly higher levels than Group A (p<0.001 for all comparisons). These findings indicate a progressive increase in salivary MDA levels from Group A to Group C (Table 7 and Figure 2).

**Table 7:** Tukey's post hoc test for pairwise comparison for malondialdehyde *: statistically significant Overall comparison was performed using one-way analysis of variance: F(2,72)=3.45; p=0.037. Pairwise comparisons were performed using Tukey's post hoc test.

Comparison	Mean difference	P-value
Group A vs. Group B	-15.35	<0.001*
Group A vs. Group C	-64.02	<0.0001*
Group B vs. Group C	-48.67	<0.0001*

## Discussion

Periodontitis is a chronic inflammatory disease initiated by periodontal pathogenic microorganisms and mediated by the host immune response, leading to the progressive destruction of the periodontium [15]. This immune response activates an inflammatory cascade involving key mediators such as cytokines, leukotrienes, and prostaglandins. Recently, salivary biomarkers like chemerin and MDA have gained attention for their roles in disease pathogenesis. Chemerin, a pro-inflammatory adipokine, influences immune cell recruitment, while MDA, a byproduct of oxidative stress, reflects lipid peroxidation and tissue damage [16,17]. Increased levels of these biomarkers correlate with the severity of periodontal inflammation. Importantly, both chemerin and MDA are also related to OSCC, as long-term inflammation and oxidative stress are known contributors to carcinogenesis [18]. Their increased salivary levels in OSCC patients suggest potential as dual-purpose biomarkers for distinguishing between inflammatory and malignant oral conditions. Given its non-invasive nature and molecular richness, saliva serves as a valuable diagnostic medium, enhancing early detection, disease monitoring, and treatment assessment in both periodontal disease and OSCC [19,20].

In this study, Group B participants were older. They showed greater periodontal destruction than Groups A and C, supporting reports by AlJehani [21] linking age to periodontitis and Singh et al. [22] identifying older age as a risk factor for OSCC. Gender distribution was similar across groups (p=0.94), ruling out gender as a confounding factor.

Our research reveals that patients with periodontitis and OSCC exhibit higher periodontal indices, along with increased levels of oxidative and inflammatory biomarkers. This aligns with current literature that associates poor oral hygiene with oral health issues. For instance, Bakdash recognized inadequate oral hygiene as a significant contributor to periodontal disease [23], and Rosenquist et al. identified it as an independent risk factor for OSCC [24].

Lertpimonchai et al. noted that fair to poor oral hygiene increases the risk of periodontitis by two to five times, aligning with our results [25]. Additionally, Farquhar et al. suggested that oral health influences tumor biology through inflammation, supported by our findings of elevated biomarkers in OSCC patients [26]. Mello et al. emphasized that poor oral hygiene worsens carcinogenic effects, suggesting that compromised periodontal health may promote OSCC through inflammatory and oxidative pathways [27].

The current study observed a progressive increase in salivary chemerin levels from healthy individuals (Group A: 23.89 ng/mL) to periodontitis patients (Group B: 26.18 ng/mL), with the highest levels in OSCC patients (Group C: 38.63 ng/mL). ANOVA test revealed significant differences among the groups (p<0.05). Chemerin, known for its roles in inflammation, is associated with chronic conditions like periodontitis, as supported by findings from Özcan et al. and Abdulmajeed and Mahmood, who linked elevated chemerin to periodontal tissue damage [28,29]. Wang et al. and Abood and Aldhaher also advocated for chemerin as a biomarker for inflammatory periodontal conditions [30,31]. 

Furthermore, previous studies, including Ghallab and Shaker and Malhotra et al., also reported elevated salivary chemerin levels in OSCC, supporting its potential as a diagnostic marker for early oral cancer detection [32,33]. Susha and Ravindran emphasized chemerin's role in tumor progression via immune modulation [34].

The mean salivary MDA levels consistently increased from Groups A to C, validating its role as an oxidative stress marker. Khalili and Biloklytska and Wang et al. found elevated MDA levels correlating with periodontal disease severity [35,36]. Cherian et al. and Gupta et al. highlighted MDA's relevance in oxidative stress during periodontal inflammation [37,38]. Our findings align with Shetty SR et al. and Wiśniewski et al. regarding MDA as a reliable biomarker in oral cancers [39,40].

Pearson's correlations showed moderate positive correlations between salivary chemerin and clinical periodontal parameters, though not statistically significant (p>0.05). Although one-way ANOVA revealed a statistically significant difference in salivary MDA levels among the groups (p=0.037), the overall effect size was modest, likely reflecting intra-group variability. However, post hoc analysis demonstrated clear and consistent pairwise differences between all groups. This suggests a structured, stepwise increase in oxidative stress across disease severity groups, with higher MDA levels observed in more advanced conditions. The combined interpretation of ANOVA and post hoc findings indicates a biologically meaningful trend despite moderate overall variance. A significant correlation (r=0.996; p=0.036) emerged between chemerin and MDA, suggesting an interaction between inflammation and oxidative stress in the pathogenesis of periodontal disease. This study supports the potential of chemerin and MDA as salivary biomarkers for diagnosing and monitoring both periodontal disease and OSCC, with the non-invasive nature of saliva collection offering substantial benefits for screening, especially in resource-limited settings.

The study has several limitations, including a smaller sample size, which could restrict the statistical power and the ability to generalize the findings to the wider population.

## Conclusions

This research revealed significantly increased levels of salivary chemerin and MDA in individuals with periodontitis and OSCC, with the highest concentrations observed in OSCC patients. This indicates a gradual rise in these biomarkers from chronic inflammation to cancerous transformation. The noticeable correlation between chemerin and MDA points to a common underlying mechanism that involves oxidative stress, immune system modulation, and tissue damage. These results underscore the promise of chemerin and MDA as non-invasive salivary biomarkers for the early detection, risk evaluation, and monitoring of disease progression in periodontal disease and oral cancer. There is a need for further extensive, long-term, and molecular studies to validate their clinical relevance and to create dependable saliva-based diagnostic tools for high-risk groups.
